# Evaluation of epidemiological, clinical, and microbiological features of vulvovaginal candidiasis

**DOI:** 10.3205/dgkh000544

**Published:** 2025-04-30

**Authors:** Reza Faraji, Abbas Maleki, Abbas Gheisoori, Taha Rashidi, Amirhossein Salimi Mansouri, Fatemeh Rashidi, Sadegh Faraji, Alireza Kashefizadeh, Arezoo Bozorgomid

**Affiliations:** 1 Tuberculosis and Lung Diseases Research Center, Ilam University of Medical Sciences, Ilam, Iran; 2 Clinical Microbiology Research Center, Ilam University of Medical Sciences, Ilam, Iran; 3 Student Research Committee, Kermanshah University of Medical Sciences, Kermanshah, Iran; 4 Department of Clinical Science, Faculty of Veterinary Medicine, Razi University, Kermanshah, Iran; 5 Faculty of Dentistry, Kermanshah University of Medical Sciences, Kermanshah, Iran; 6 Shahid Labbafinejad Hospital, Shahid Beheshti University of Medical Sciences, Tehran, Iran; 7 Medical Biology Research Center, Health Technology, Kermanshah University of Medical Sciences, Kermanshah, Iran

**Keywords:** vaginal candidiasis, diabetes mellitus, women

## Abstract

**Background:**

Vaginal candidiasis is induced by abnormal growth of yeast on the mucous membranes of the female genital tract. Approximately 75% of women experience a yeast infection once in their lifetime. This study explored the epidemiological, clinical, and microbiological features of vaginal candidiasis in diabetic women referred to health and treatment centers in Kermanshah in 2023.

**Methods:**

This cross-sectional descriptive study was conducted on 215 diabetic women. A questionnaire was prepared for each participant. The samples were examined microscopically and cultured on Sabouraud dextrose agar (SDA). To identify different species of *Candida* (C.), various complementary tests were performed, such as the germ tube and differential sugar absorption test (API). A sensitivity test was applied to positive samples by the broth macrodilution method. Data were analyzed using the chi-squared test in SPSS.

**Results:**

Out of the 215 vaginal swabs investigated, 66 specimens were *Candida*-species positive (30.7%). 11.6% of participants were diagnosed with candidal vulvovaginitis by direct microscopic examination and 20.9% by culturing on SDA. The *Candida* species isolated were: *C. albicans* with 36 cases (54.5%), *C. glabrata* with 14 cases (21.2%), *C. tropicalis* with 9 cases (13.6%) and *C. parapsilosis* with 7 cases (10.6%). All species isolated showed the same sensitivity to the antifungal drugs used.

**Conclusion:**

The culture method was more sensitive than the direct microscopic examination. *C. albicans* was the most prevalent species isolated from patients. Non-albicans species were not prevalent.

## Zusammenfassung

**Hintergrund:**

Die vaginale Candidiasis wird durch das abnormale Wachstum von Hefepilzen auf der Schleimhaut des weiblichen Genitaltrakts hervorgerufen. Ungefähr 75% der Frauen erleiden einmal im Leben eine Hefepilzinfektion. Diese Studie untersuchte die epidemiologischen, klinischen und mikrobiologischen Merkmale der vaginalen Candidiasis bei diabetischen Frauen, die im Jahr 2023 an das Gesundheits- und Behandlungszentren in Kermanshah überwiesen wurden.

**Methode:**

Die deskriptive Querschnittsstudie wurde an 215 Diabetikerinnen durchgeführt. Für jede Teilnehmerin wurde ein Fragebogen erstellt. Die Proben wurden der mikroskopischen Untersuchung und Kultur auf Sabouraud-Dextrose-Agar (SDA) unterzogen. Ergänzende Tests wurden durchgeführt, um verschiedene Candida-Arten zu identifizieren, wie z.B. Keimschlauchtest und Zuckerabsorptionstests (API). Der Sensitivitätstest wurde auf die positiven Proben mittels der Bouillon-Makrodilutionsmethode angewendet. Die Daten wurden mit SPSS unter Verwendung des Chi-Quadrat-Tests analysiert.

**Ergebnisse:**

Von 215 untersuchten Vaginalabstrichen waren 66 Proben positiv auf Candida-Spezies (30,7%). Bei 11,6% wurde durch direkte mikroskopische Untersuchung, bei 20,9% durch Kultivierung auf SDA eine Candida-Vulvovaginitis diagnostiziert. Die isolierten Candida-Arten waren: *Candida (C.)* *albicans* mit 36 Fällen (54,5%), *C. glabrata* mit 14 Fällen (21,2%), *C. tropicalis* mit 9 Fällen (13,6%) und *C. parapsilosis* mit 7 Fällen (10,6%). Die Empfindlichkeit der isolierten Arten gegenüber den verwendeten Antimykotika war gleich.

**Schlussfolgerung:**

Die Kulturmethode war empfindlicher als die direkte mikroskopische Untersuchung. *C. albicans* war die am häufigsten isolierte Spezies. Allerdings kamen keine non-*C. albicans* Arten vor.

## Introduction

Diabetes is the most common endocrine disease that can affect any organ or system of the body. It affects people of all ages. Diabetic patients are at high risk for severe microvascular and macrovascular complications, such as cardiovascular disease, end-stage renal disease, and blindness [1], [2]. Furthermore, diabetic patients are more susceptible to bacterial and fungal infections, including those caused by *Candida* species [3], [4]. Vaginal candidiasis is an important problem in diabetic patients, so that it is classified by the World Health Organization as a pathological condition. This disease is caused by the abnormal growth of yeasts on the mucous membranes of the female genital tract [5], [6]. It is estimated that approximately 75% of women experience this infection at least once in their lifetime [7], [8]. In addition to diabetes, several factors such as previous colonization by yeast, immunodeficiency disorders, pregnancy, use of wide-spectrum antibiotics, high-dose oral contraceptives, obesity, and drug addiction increase vaginal candidiasis in women [1], [5], [6], [8], [9], [10], [11]. Clinical symptoms of vaginal candidiasis are characterized by severe itching of the vulva, leukorrhea, dyspareunia, heartburn, edema, and vulvovaginal erythema. Early and reliable diagnosis of clinical yeast pathogens at the species level is important in choosing an effective treatment. Antifungal agents are used to treat vaginal candidiasis [5]. Further, 85–90% of the causative agent of vaginal candidiasis,* Candida (C.) albicans*, followed by *C. glabrata*, *C. tropicalis*, and other species such as* C. parapsilosis* and *C. krusei* are involved in vaginal candidiasis infection to a lesser extent [12]. This study explored the epidemiological, clinical, and microbiological features of vaginal candidiasis in diabetic women referred to health and treatment centers in Kermanshah in 2023.

## Materials and methods

This descriptive cross-sectional study used a non-interventional sampling method to select samples randomly. Sampling was performed on 215 diabetic women referred to the health and treatment centers of Kermanshah from April 2023 to March 2024. The inclusion criterion was affliction with diabetes defined according to World Health Organization guidelines as a fasting blood sugar (FBS) of 140^+^ mg/ml. All participants provided informed written consent for the study. A questionnaire was prepared for each patient, which provided information about age, education level, occupation of self and husband, vaginal clinical symptoms, contraceptive methods, sexual relations, duration of diabetes, type of diabetes, and blood sugar levels. Two sterile swabs were collected from vaginal secretions, one swab for direct microscopic examination and one swab for culture. To perform direct microscopic examination, a direct slide was prepared from each of the samples, fixed with flame heat, stained with methylene blue, and examined under 40x magnification. The presence of yeasts, yeast-like cells, or hyphae (with or without septate) was evaluated under the microscope. The other swab was cultured in a plate containing Sabouraud dextrose agar containing 50 mg of chloramphenicol; the patient number and culture date were written on the plates which were kept at 30°C for 48–72 h. Colony growth in the culture medium was reported as a positive sample. Subsequently, the API test was used to detect *Candida* spp. (API 20c AUX kit, Biomerieux) and yeast species was detected from the special table attached to the kit. After determining the *Candida* spp., the minimum inhibition concentration (MIC) was determined by the broth macrodilution method (a series of 11 test tubes) according to NCCL M 27A instructions [13]. The 72-hour culture of the tested yeast in a sterile physiological serum was used to prepare a suspension equivalent to 0.5 McFarland turbidity. Ten dilutions from 0.125 to 64 µg/mL were prepared from 3 drugs, including amphotericin B, fluconazole, and clotrimazole. One tube was used as a positive control (without antibiotics). After 24 h, the series of tubes was visually checked for turbidity, indicating the growth of yeasts. The minimum concentration at which the yeast did not visibly grow was determined as MIC [12], [14]. The collected data were analyzed using the chi-squared test in SPSS (P<0.05). 

## Results

**Figure 1:**
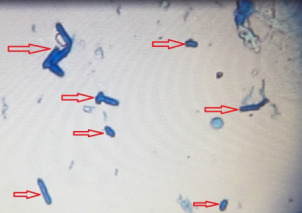
Microscopic image of pseudohyphae (40-fold magnification)

**Figure 2:**
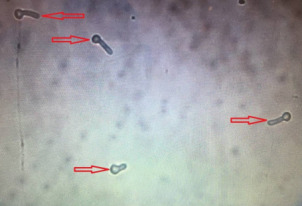
Microscopic image of germ tube (40-fold magnification)

**Figure 3:**
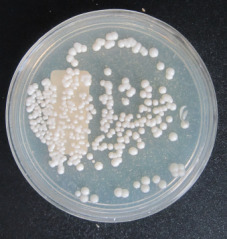
Candida colony growth in Sabouraud dextrose agar medium

215 diabetic women were examined in the 12-month period. The age of the studied women ranged between 20 and 70 years (mean 52±9 years). The mean level of fasting blood sugar in these patients was 190±65 mg/dL. Five (2%) patients had type 1 diabetes and 210 (98%) had type 2 diabetes. 66 specimens were *Candida* spp. positive (30.7%). The prevalence of vaginal candidiasis was reported as 11.6% by direct microscopic examination (Figure 1 (Fig. 1) and Figure 2 (Fig. 2)) and 20.9% by culture (Figure 3 (Fig. 3)).

The isolated species were *C. albicans* (54.5%), *C. glabrata* (21.2%), *C. tropicalis (13.6%), and C. parapsilosis* (10.6%), of which *C. albicans* is the most dominant species causing the disease (Table 1 (Tab. 1)). Moreover, 43.9% of the affected women were housewives and the rest were employed. In terms of education, 83.3% of the affected women had an illiterate and high school, and 16.7% were university graduates. Also, 85% of the patients had employed husbands. The education of 74% of the husbands of the respondents was at the level of high school or below. Further, 50% of women had used intravaginal contraceptive devices such as IUD and 50% had used extravaginal contraceptive devices such as condoms, or birth control pills. Additionally, 70% of the patients possessed risk factors for the disease, including obesity, kidney and liver disease, pregnancy, long-term use of antibiotics and corticosteroids, wearing tight nylon clothes, and sexual intercourse. In patients with pain, itching and white discharge, microscopic studies and cultures were performed. 58 (87.9%) patients had white discharge, 42 (63.69%) patients had pain, and 45 (68.2%) patients had itching. The above symptoms were observed in most of the patients, and the rest of these complaints were either unrelated to vaginal candidiasis or pertained to other types of vaginitis. The chi-squared test revealed no statistically significant correlation between vaginal candidiasis and the occupation of affected women and their husbands, husband’s education, and contraceptive methods, and none of these factors were related to a positive Candida culture. However, the relationship between age, education level, disease risk factors, and type of vaginal symptoms with candidal vulvovaginitis was significant (P=0.000) (Table 2 (Tab. 2)). 

Moreover, the chi-squared test suggested no statistically significant correlation between the sensitivity of the different isolated *Candida* spp. to amphotericin B, fluconazole, and clotrimazole (P=1.000) (Table 1 (Tab. 1)).

## Discussion

Vaginitis is a global problem for millions of women around the globe. Vulvovaginal candidiasis is defined as signs and symptoms of inflammation of the vulva and vagina with the presence of *Candida* spp. It has been found that when there is a change in the host environment, *C. albicans* can become a disease-inducing pathogen [15], [16], [17]. In this study, among 215 diabetic women, the prevalence of vaginal candidiasis by direct microscopic examination and culture was 11.6% and 20.9%, respectively. In the study by Al Halteet et al. [5], the prevalence of vaginal candidiasis was 12.7% by direct microscopic examination and 17.5% by culture. They reported the sensitivity and accuracy of direct microscopic examination as 81.3% and 100%, respectively; both sensitivity and accuracy were 100% for the culture method. In the study by Yokoyama et al. [1] that used the culture method, the prevalence of vaginal candidiasis was reported as 14.9%. In the studies by Krishnasamy et al. [15], Kumari et al. [16], and Namrata Kalia et al. [18], the prevalence of vaginal candidiasis was reported as 35%, 30.6%, and 47%, respectively, using the culture method. The results of this research and other similar studies show that the secretion culture method is more sensitive than the microscopic examination. In this regard, Al Halteet et al. [5] stated that the conventional culture method was more valid than other methods for diagnosing vaginal candidiasis, and the culture of vaginal secretions seems necessary for a definite and final diagnosis. Increased resistance to antifungals leads to treatment failure; thus, identification of *Candida* spp. seems mandatory for better treatment and prevention of drug resistance [15]. In this study, the most frequent species isolated from patients was *C. albicans* (54.5%). As in our study, in the study by Al Halteet et al. [5], *C. albicans* was identified as the most prevalent species with 59.3%. Additionally, in the studies by Krishnasamy et al. [15] and Noori et al. [19], *C. albicans* was the most frequent species with 25% and 66%, respectively. Unlike our study, however, Emeribe et al. [20] found the prevalence of non-albicans species (7.5%) to be greater than that of *C. albicans*. (6.5%). Moreover, in the study by Aring et al. [21], non-albicans species dominated, with *C. glabrata* making up 10.5%, which was more than *C. albicans*. Different reasons for the prevalence of albicans or non-albicans species have been mentioned in other studies [15], [22], having to do with the first step in the emergence of a yeast infection being the adherence of the yeast to the vaginal mucus. On the one hand, in diabetic patients, hyperglycemia causes excessive and abnormal growth of *Candida*. On the other hand, *C. albicans* seems to be stickier than non-albicans spp. This issue can be considered as one of the possible reasons for the dominance of this species over non-albicans spp. [15], [22]. Nonetheless, in their study on single-dose antifungal treatment, low-dose azole maintenance regimens, and the use of over-the-counter (OTC) antifungal drugs, Emeribe et al. [20] considered these to be among the possible reasons for the predominance of non-albicans spp. over *C. albicans*. Noori et al. [19] stated that the prevalence of different *Candida* spp. can be due to differences in geographic regions, sexual behaviors, cultures, customs of different nations, as well as differences in study design, target population, and diagnostic methods. In our study, a statistically significant difference was observed between age and the prevalence of vaginal candidiasis, and most cases of vaginal candidiasis were seen in the age group <40 years. Similar to our study, Noori et al. [19] found a statistically significant difference between the prevalence of vaginal candidiasis and age, so that the age group of 27–35 years had the highest affliction rate. Furthermore, in the study by Alo et al. [23], women aged 36–40 years had the highest rate of candidiasis. The reasons may be the higher sexual activity of this age group, physiological and hormonal changes, increased vaginal secretions, and the use of different contraceptive methods [19]. However, in the study by Emeribe et al. [20], there was no statistically significant difference between the prevalence of vaginal candidiasis and age. In our study, a statistically significant correlation was found between educational level of the patients and the prevalence of vaginal candidiasis; the absence of vaginal candidiasis can be attributed to the knowledge of favorable conditions for the disease, and compliance with hygiene, etc. In our study, a direct correlation was observed between vaginal clinical symptoms and prevalence of vaginal candidiasis, so that 87.9% of the patients exhibited white discharge, 63.6% had pain, and 68.2% had itching. In the study by Krishnasamy et al. [15], 32% of women had white discharge, 28% showed itching, and 11% sustained pain. Latha Ragunathan et al. [24] found that 31% had itching, 29.4% had white discharge, and 15.6% had pain. In contrast, Peer [25] showed that 25.4% of patients with vaginal candidiasis had no clinical symptoms. It follows from the above that vaginal candidiasis is not always associated with severe and obvious symptoms; rather, in some cases, they have no symptoms or possibly mild symptoms. In our study, 50% of women used intravaginal contraceptive devices, such as an IUD, and 50% used extravaginal contraceptive devices such as condoms and birth-control pills. In the study by Ocak et al. [26], the prevalence of *Candida* spp. was about 15% in women who received oral contraceptive pills, 12% in women who wore an intrauterine contraceptive device, and 6% in women who did not use any contraceptive method. The most likely hypothesis is that both antibiotics and contraceptives can alter the vaginal microbiota and indirectly cause fungal infections [27]. In our study, 70% of the patients had risk factors for the disease, including obesity, renal and hepatic disease, pregnancy, long-term use of antibiotics and corticosteroids, wearing chafing nylon clothes, and coitus, which is similar to previous studies [22], [26], [28], [29], [30], [31], [32], [33], [34], [35], [36]. In our study, there was no statistically significant correlation between the sensitivity of *Candida* spp. isolated from patients to three antifungal drugs: amphotericin B, fluconazole, and clotrimazole. In the study by Al Halteet et al. [5], the resistance rates of *C. albicans*, *C. glabrata*, and *C. krusei* were 11.1%, 0.0%, and 100%, respectively, indicating that *C. glabrata* is more sensitive to fluconazole than the other species. Finally, in the study by Goswami et al. [14], the most dominant species, *C. glabrata*, had the highest resistance to fluconazole with 67.1%. This can be due to many factors, including previous antifungal drug exposure, resistance of genes, improved membrane lipid fluidity and asymmetry, involvement of other pharmacotherapy drugs, and intrinsic resistance of *Candida* spp. [5]. 

## Conclusion

*C. albicans* was the most frequent species isolated from patients. According to different results obtained from the sensitivity of *Candida* spp. to antifungal drugs in various studies, it can be said that the sensitivity of *Candida* spp. to antifungal drugs is variable and causes problems in treatment. Consequently, it is suggested that for proper treatment, identification of the *Candida* spp. and the sensitivity of the isolated species to antifungal drugs be taken into consideration, with treatment carried out accordingly.

## Notes

### Authors’ ORCID

- Reza Faraji: 0000-0002-5973-7301
- Arezoo Bozorgomid: 0000-0003-2093-9317

### Ethical approval 

The protocol was approved by the Ethics Committee of Kermanshah University of Medical Sciences (KUMS.REC.1394.349).

### Funding

None.

### Acknowledgements

We hereby express our gratitude and appreciation to Kermanshah University of Medical Sciences and the health and treatment centers of Kermanshah, which cooperated fully in conducting this research.

### Competing interests

The authors declare that they have no competing interests.

## Figures and Tables

**Table 1:**
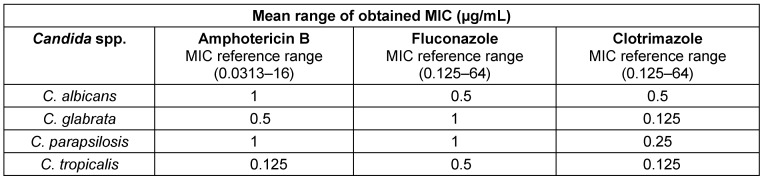
Sensitivity of *Candida* spp. isolated from affected women to the tested antifungal drugs

**Table 2:**
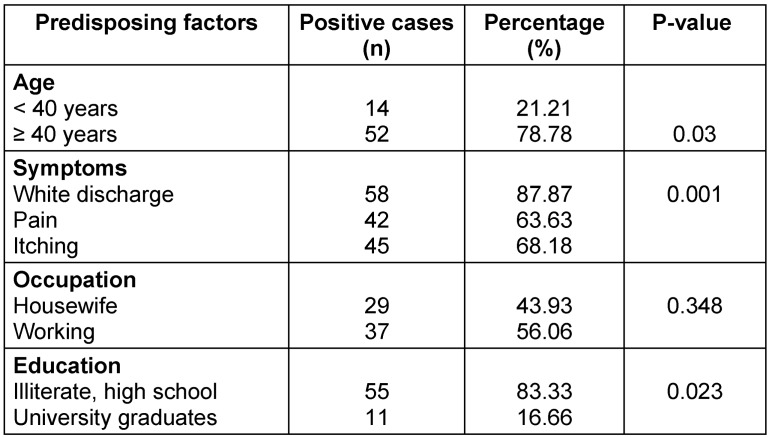
Absolute and relative frequency distribution of affected women in terms of variety of influencing factors
